# Case of Fatal Diarrhæa

**Published:** 1804-05-01

**Authors:** 


### *To the Editors of the Medical and Physical Journal.*

GENTLEMEN,

A Reader, not of the Faculty, will be much obliged to any gentleman who, in your Journal, will communicate the cause of the illness of a fine little boy, within a fortnight of two years of age, lost the beginning of January last.

Tuesday the 3d, he had gone down stairs by himself, clothed in a great coat against cold, to look for his breakfast, but soon returned without it. The morning was cold and a little frosty. The same morning, before nine o'clock, he ate a good breakfast as usual; but, about ten, he came to look for his mother, seemingly cold, and expressing pain, cold, or uneasiness, by a whine. He had previously discharged a considerable quantity of purgative, slimy sort of stool: from that time he was kept quiet and in the lap. At his dinner time he refused his usual pudding, and through the evening would take nothing but tea or drink. His sleep at night was very unquiet: some rhubarb in currant jelly was given to him; after which he seemed somewhat easier.

In the morning of Wednesday the 4th, a gentleman of the faculty saw him, and sent some saline mixture, calomel, and a blister, the size nearly of a man's hand, for his stomach. His stools had become greenish; the fever, during the day, awaked him with a start whenever he fell asleep, and the case was considered and treated as bilious. At night he moaned for hours, and was in a state of fever with scorching heat; the blister was on him and had risen. The dejections were still greener.

Thursday the fever was considerably abated. He slept much, seemingly comatose. His apothecary agreed to have a physician sent for, who came, but not till past the middle of the next day. He had taken nothing but a very little tea during his illness, and had become very weak, wasted, and emaciated, with sunken and hollow eyes.

Friday, three in the afternoon, the physician saw him, and observed his very laborious breathing, which happened then, but had been only occasional, and afterwards went off. His dejections being green, calomel was prescribed, and a blister for the inside of each leg above the ancle. His life was not expected. But,

Saturday morning, although during the preceding night the the hiccough had been often heard, he seemed a little revived, and rather to enjoy gruel. Dejections were not quite so green; debility increased; his sleep, coma.

Sunday morning, about three o'clock, he was convulsed, seemingly with approaching death; and, after several fits, expired soon after six o'clock.

During most of his short illness, he continually rubbed his nose with his left hand. His habit previously to his illness had been rather plethoric; but he had been otherwise healthy and strong, excepting an occasional weaknesss when on his legs, from teeth, as was judged: he had sixteen perfectly out, and was then cutting the last four at each of the extremes of either jaw. He had no cough of any seeming consequence, nor cold; unless it had been taken in the bowels the preceding Monday or Tuesday morning, by a sudden change of the weather from warm to frosty; or by some momentary and unobserved exposure to the cold air.

His diet had been plentiful, but not gross. No butter, very little meat, no beer; very little good port wine, occasionally.

The weather had for some weeks prevented his being much taken out, but he was not confined to warm rooms; ran about a hall with the door open; and, when fine and clear enough, upon a gravel walk in a garden.

An answer to this case by any Correspondent, or a reference to any book or treatise, *De præcipuis prope biennium infantum, morbis, & eorum causis,* will much oblige,

*April* 10, 1804. ACADEMICUS.

